# Incidental finding of a congenital left ventricular diverticulum

**DOI:** 10.21542/gcsp.2024.50

**Published:** 2024-12-31

**Authors:** Ibrahim Oumarou Hamissou, Nicolas Hugues, Emeric Lager, Romain Pastre

**Affiliations:** 1 Department of Cardiology, University Mohammed-V of Rabat, Morocco; 2 Interventional Cardiology Department, Emile Roux Health Care Center, Le Puy-En-Velay, France

## Abstract

Left ventricular diverticulum is a rare abnormality for which diagnosis is often delayed due to its asymptomatic character. Multimodal imaging can help in diagnosis of this potentially confusing condition. We present a 48-year-old female patient with a medical history of a paradoxical thromboembolic events who benefited from the closure of a patent foramen oval in another center. She presented a year later at our department for a lower back pain where an incidental finding of a congenital left ventricular diverticulum was made on a contrasted computed tomography scan.

## Introduction

Left ventricular diverticulum is defined as an outpouching from the ventricle with a preserved synchronous contractility^1^. Most cases are discovered incidentally on imaging studies performed for other medical conditions, but can also be revealed by a thromboembolic event. The diagnostic suspicion is made on echocardiography and is further confirmed by other complementary investigations, having ruled out a left ventricular aneurysm which is the main differential diagnosis. There are currently no recommendations for the management of left ventricular diverticulum. In this case report, we discuss our diagnostic approach using multimodal imaging and the specific management of our patient.

## Case presentation

### Medical history

The patient was a 48-year-old woman with moderate obesity (BMI 34.54 kg/m^2^), who stopped smoking several years ago. Her surgical history includes a myomectomy for uterine fibroids and a childhood appendectomy. In September 2021, she underwent closure of a patent foramen ovale (PFO) at another facility following a renal infarction caused by thromboembolism. Post-procedure, she was prescribed dual antiplatelet therapy consisting of aspirin 75 mg daily for five years and clopidogrel 75 mg daily for three months.

### History of presenting complaint

**Figure 1.**
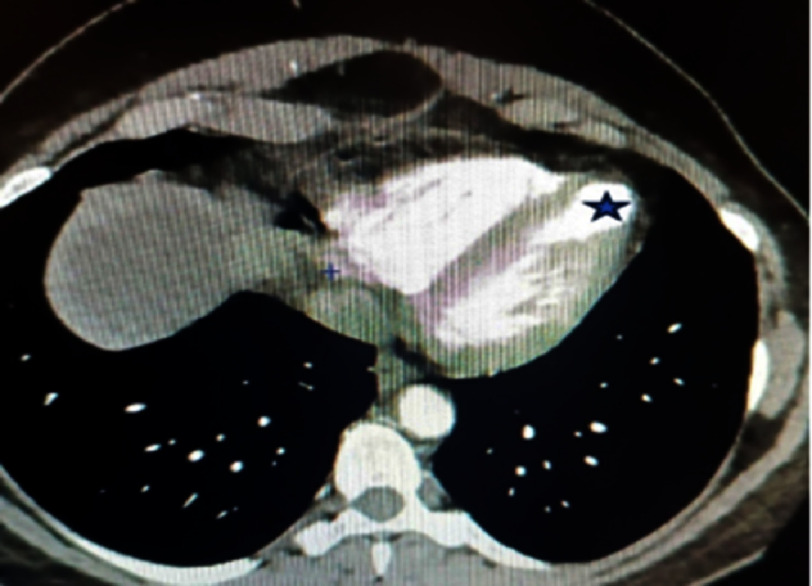
Aspect of left ventricular apical aneurysm (star) in a contrasted chest computed tomography scan.

One year later (June 2022), she consulted our department about a lower back pain. A recurrence of a thromboembolic event was suspected and we performed a thoraco-abdominopelvic computed tomography scan that found no peculiarities in the abdominal region, but the thoracic scans showed an aspect of an apical aneurysm of the left ventricle (Figure 1).

### Investigations

**Figure 2.**
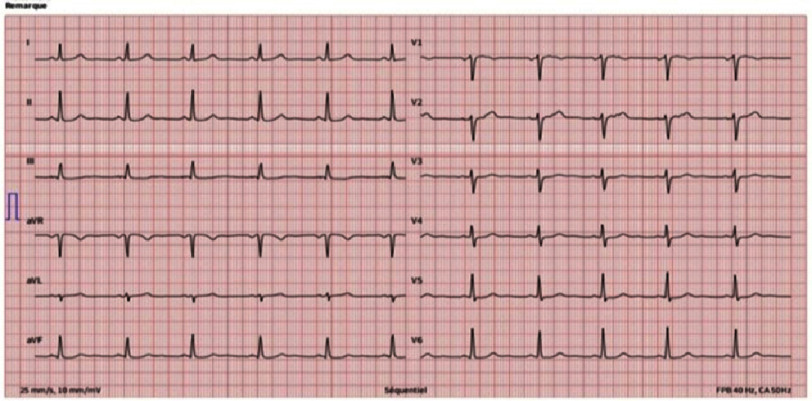
12-lead ECG in sinus rhythm.

The patient had no cardio-pulmonary symptoms, the heart sounds were normal with no murmurs, there were no signs of heart failure and his electrocardiogram registered a sinus rhythm (Figure 2).

**Figure 3.**
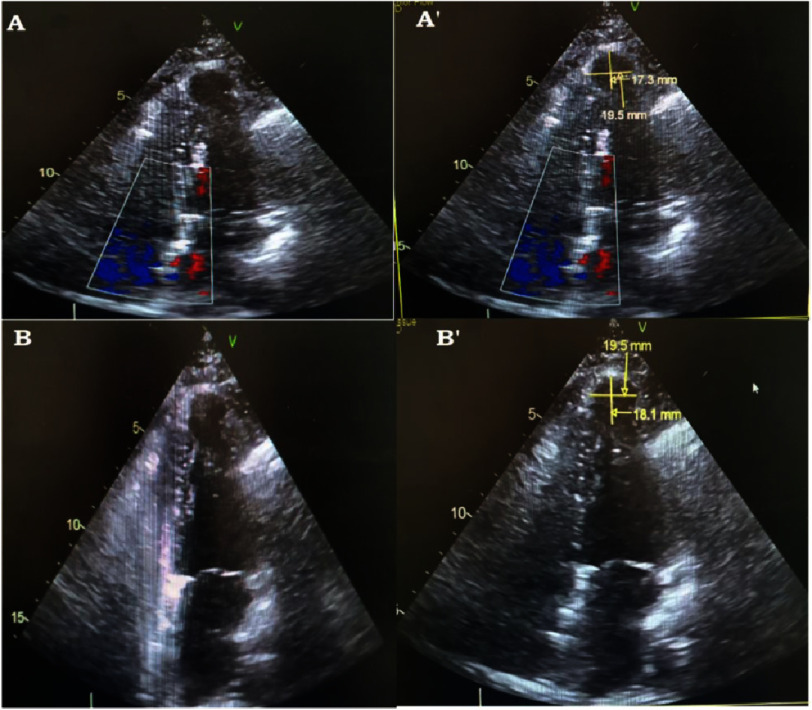
Contrasted echocardiography showing apical aneurysm aspect of the left ventricle measuring 17.3 mm × 19.5 mm in apical four chamber (A’) and 19.5 mm × 18.1 mm in apical two chamber (B’).

A trans-thoracic echocardiogram performed on the day of admission revealed an apical aneurysm aspect measuring 17.3 mm × 19.5 mm in an apical four-chamber view and measuring 18.1 mm × 19.5 mm in an apical two-chamber view (Figure 3).

**Figure 4.**
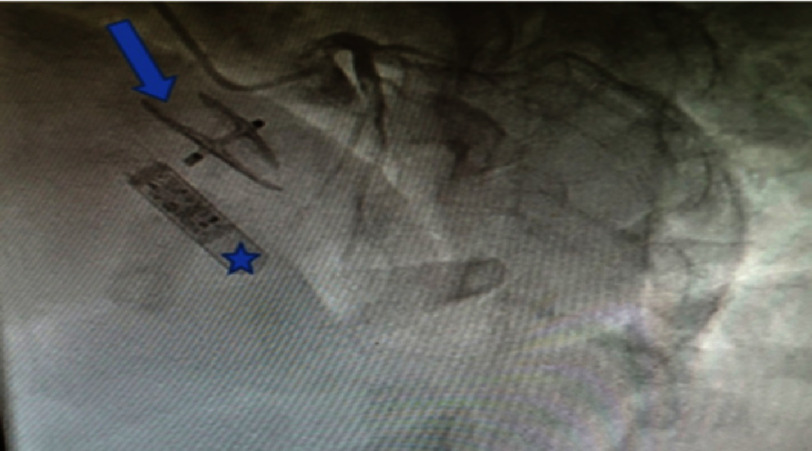
Caudal right anterior oblique view, showing PFO closure prosthesis AMPLATZER 25 ×18 mm (arrow) and the implanted loop recorder (star).

An exploration the next day by a trans-esophageal echocardiography showed no residual shunt at the PFO closure site. There was no presence of thrombus in the left auricle, and the left ventricle ejection fraction was 65%. An implantable loop recorder (Figure 4) was placed for six months, from June to December 2022, and it did not detect any atrial fibrillation episodes or other emboligenic cardiac arrhythmia.

The patient underwent thrombophilia testing and supra-aortic Doppler imaging to investigate potential sources of embolism. Both yielded normal results.

After these first investigations, two diagnostics hypotheses were considered regarding the left ventricular aneurysmal aspect: an ischemic apical aneurysm of the left ventricle or a congenital left ventricular apical diverticulum.

**Figure 5.**
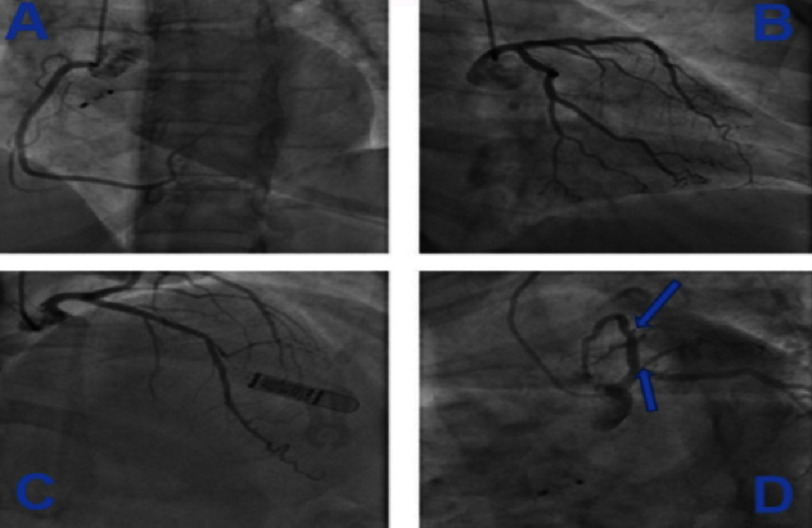
(A) Left anterior oblique view showing right coronary artery. (B) Caudal right anterior oblique view showing circumflex artery. (C) Cranial right anterior oblique view showing LAD. (D) Caudal left anterior oblique view showing non-significant lesion.

This required further investigations. First a coronary angiography (December 2022) found non-significant atheromatous lesions of about 40% on the left anterior descending artery (LAD) and on the ostium of the ramus intermedius artery (Figure 5).

**Figure 6.**
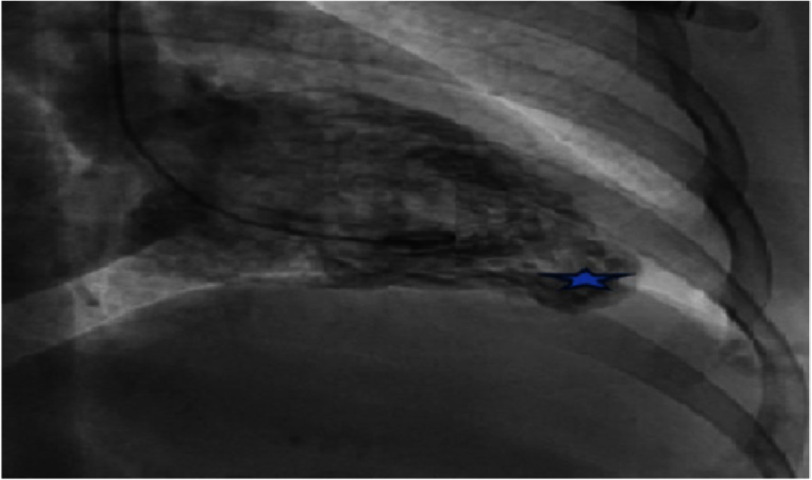
Left ventricle apical diverticulum on ventriculography.

Left ventriculography showed the presence of an accessory chamber at the apex of the left ventricle, evoking in the first instance a left ventricular apical diverticulum (Figure 6).

**Figure 7.**
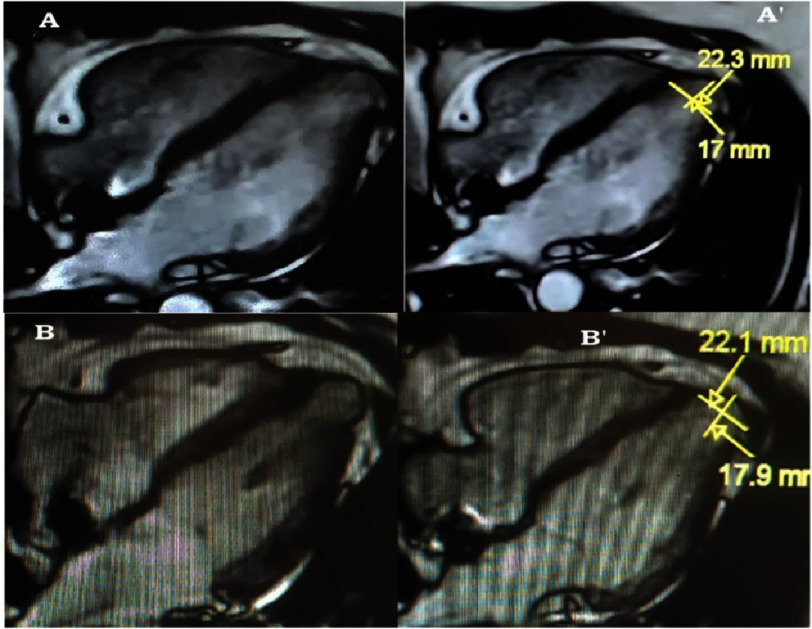
Left ventricle apical congenital diverticulum on myocardial MRI on apical 4 chambers view in systole (A and A’); diastole (B and B’;) measuring 22.3 mm × 17 mm (A’) and 22.1 mm × 17.9 mm (B’).

Diagnosis was confirmed a month later (January 2023) by an anatomical myocardial MRI, suggesting first a left ventricular apical congenital diverticulum (star), with a normal contraction and measuring 17 mm × 22 mm. Note that gadolinium was not injected due to patient claustrophobia and fear of enclosure in the MRI machine (Figure 7).

### Management

Between the cardiologist, neurologist and radiologist we decided to place the patient on a long-term anticoagulant therapy based on Non-vitamin K oral anticoagulants (Apixaban 5 mg, twice daily). This choice of NOAC therapy was based on the patient’s high thromboembolic risk profile, and aspirin was discontinued.

## Discussion

Left ventricular diverticulum is a rare congenital abnormality in approximately 0.1% cases of congenital heart disease. Some authors have reported an incidence of 0.4%, such as 3 of 750 cases following autopsy after cardiac deaths. According to a meta-analysis, 453 left ventricle diverticulum cases were described from 1816 to 2022. The etiopathogenesis is due to partial cessation of ventricular wall formation during the 4th week of the embryonic period^2^.

Around 30% of cases of left ventricle diverticulum are isolated^3^ and 70% are associated with other cardiac malformations (ventricular septum defect, coronary anomalies, atrial septum defect, ectopic cordis)^4^ or extracardiac abnormalities such as Pentalogy of Cantrell, which includes: supra umbilical hernia, hernia of the anterior part of the diaphragm, sub-sternal malformation and diverticulum of the left ventricle.

The diverticulum of the left ventricle is asymptomatic and most often discovered incidentally during imaging performed for other reasons. However, several examinations are needed to form a complete diagnosis.

Echocardiography shows an accessory anechoic chamber from left ventricle lateral wall, that remains contractile and synchronous with the left ventricle. Contrasted cardiac computed tomography helps to determine with precision, the dimensions of the diverticulum and its different localizations: mostly apical types (left ventricle apex) and non-apical types (sub-aortic, left ventricle inferior wall, right ventricle)^5^. It is also used to assess coronary arteries in case of low cardiovascular risk.

Myocardial MRI shows a normal myocardial signal with no delayed gadolinium enhancement^6^. Coronary angiography is performed in the case of a coronary artery disease suspicion and left ventriculography performed at the same time helps to describe the congenital left ventricular diverticulum lesion.

The main differential diagnosis is the left ventricular aneurysm, characterized on myocardial MRI by an akinesia with transmural delayed gadolinium enhancement^6^ and commonly associated with coronary artery significant lesions or a coronary embolism possibility.

The most frequent complications are cardiac arrhythmias including ventricular tachycardia and ventricular fibrillation in 9.9% of the cases, the underlying mechanism is a macro-reentry, followed by thromboembolic events that represent 2.9% of cases. Diverticular rupture is rare in adults, but rather frequent in children, occurring in the first two years of life in 4.2% of cases^7^.

In our patient this apical topography is consistent with cases described in the literature. The absence of akinesia; the presence of synchronic contraction of the outpouching wall on echocardiography, MRI and ventriculography allowed us to rule out an apical ischemic aneurysm of left ventricle. Moreover, coronary angiography found no significant lesions. A combination of medical staff was necessary to study the balance of thromboembolic risk and the risk of diverticular rupture. Given her age and the previous history of renal infarction, we initiated long-term anticoagulation with non-vitamin K oral anticoagulants (Apixaban 5 mg, twice daily). Furthermore, our patient presented PFO, an association with a congenital left ventricular diverticulum that is infrequently documented in the literature (1% of cases). Although there is no known embryological connection between the two, this combination could potentially increase the risk of embolic events^8^.

We opted for a conservative management approach for our patient due to the following factors: the diverticulum was not excessively large, there were no complications such as heart failure, and no other indication for cardiac surgery. Most research recommends conservative treatment in similar cases, and the risk of rupture is lower in adults compared to children^7,9^. The planned follow-up included clinical exam, electrocardiogram and echocardiogram to monitor for complications such as heart failure, sustained arrythmia or an increasing size of the diverticulum over time that can lead to surgery. There is no recommendation regarding family screening to date, due to the rarity of this case and no clear genetic studies.

### What have we learned?

• Left ventricular diverticulum is a rare congenital anomaly which is mostly asymptomatic and discovered as an incidental finding on imaging studies.
• The main differential diagnosis to be considered is left ventricular aneurysm.
• While suspecting a congenital left ventricular diverticulum, imaging studies such as myocardial MRI, coronary angiogram and ventriculography may help support the diagnosis and also help to rule out a left ventricular aneurysm.
• Despite the absence of clear recommendations about the management of this rare pathology, there are some therapeutic possibilities such as medical treatment based on anticoagulation, surgery and regular follow-up.

## References

[1] Claus I, Adriaenssens B, Van Beeumen K, Timmers L, Bové T (2020). Congenital left ventricular diverticulum: A rare cause of cardiac arrhythmia. J Card Surg.

[2] Luc TQ, Hien PD, Ninh TP (2022). et al. Left ventricular diverticulum: A case report and review of the literature. Radiol Case Rep.

[3] Bayrak F, Guneysu T, Degertekin M, Gemici G (2007). Isolated left ventricular diverticulum in an asymptomatic patient [published correction appears in Eur Heart J. 29(16) (2008) 2060. Fatih, Bayrak [corrected to Bayrak, Fatih]]. Eur Heart J.

[4] Shah D, Kumar CP, Shah MS, Baraiya M (2010). Case series: Congenital left ventricular diverticulum. Indian J Radiol Imaging.

[5] Bell D, Weerakkody Y (2016). Left ventricular diverticulum. Radiopaedia.org. Radiopaedia.org.

[6] Romagnoli A, Ricci A, Morosetti D, Fusco A, Citraro D, Simonetti G (2015). Congenital left ventricular diverticulum: Multimodality imaging evaluation and literature review. J Saudi Heart Assoc.

[7] Ohlow MA (2017). Congenital left ventricular aneurysms and diverticula: an entity in search of an identity. J Geriatr Cardiol.

[8] Lapeña Reguero M, Fábregas-Casal R, Fernández-Couto M, Barge-Caballero G (2020). Congenital left ventricular outpouching and patent foramen ovale: A potentially emboligenic combination. Neurología (English Edition).

[9] Veliyev V, Sahratov H, Musayeva T (2019). Isolated congenital left ventricular diverticulum presenting as stable angina pectoris and surgical treatment. Polish Journal of Cardio-Thoracic Surgery.

